# Supplementary material for: Control of zeolite framework flexibility for ultra-selective carbon dioxide separation
Source: Nat Commun. 2022 Mar 17;13:1427. doi: 10.1038/s41467-022-29126-6 (PMC8930971; doi:10.1038/s41467-022-29126-6)
Supplement: Supplementary file 1 — Supplementary Information [file 41467_2022_29126_MOESM1_ESM.pdf]

Supplementary Information

Control of zeolite framework flexibility for ultra-selective carbon dioxide separation

Peng Du,[1] Yuting Zhang,[1] Xuerui Wang,[1] Stefano Canossa,[2] Zhou Hong,[3] Gwilherm Nénert,[4] Wanqin Jin[1] and Xuehong Gu[1,*]

[1]State Key Laboratory of Materials-Oriented Chemical Engineering, College of Chemical Engineering, Nanjing Tech University, Nanjing 211816, PR China

[2]EMAT, University of Antwerp, Antwerp 2020, Belgium

[3]Nanjing Membrane Materials Industrial Technology Research Institute Co., Ltd., Nanjing 211808, PR China

[4]Malvern Panalytical, Almelo 7600 AA, The Netherlands

P. D., Y. Z. and X. W. contributed equally.

**E-mail: xhgu@njtech.edu.cn*

Supplementary Method

The thermal expansion coefficient was defined by the following equation[1]:

$$\beta_{\mathrm{v}} = \frac{\Delta V}{V_0 \times \Delta T} \quad (1)$$

where β_{v} represents the volume thermal expansion coefficient, ΔV is the change in crystal volume, V_0 is the initial crystal volume, and ΔT is the temperature difference.

Supplementary Figures, Notes and Tables

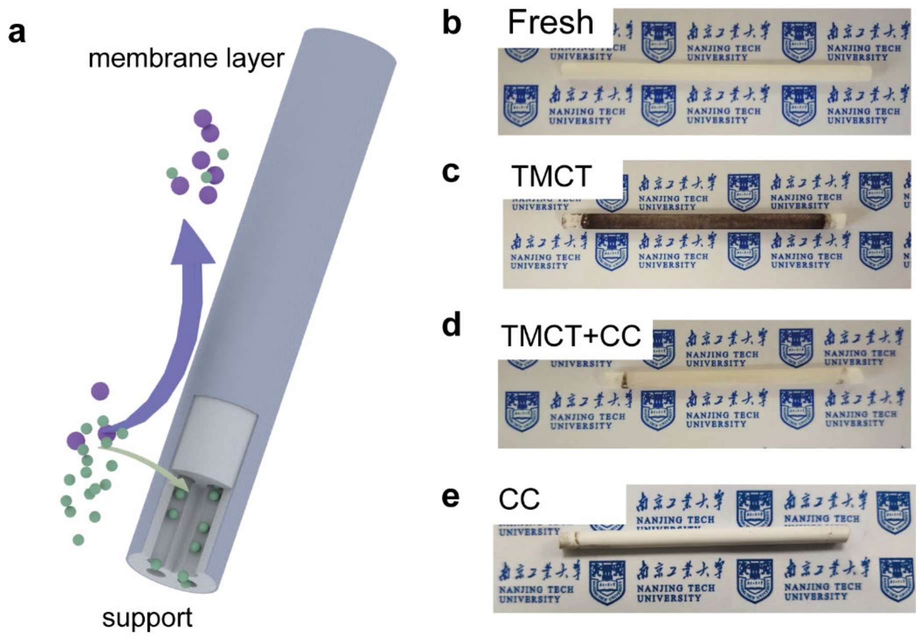

Supplementary Figure 1 The photos of 7 cm DD3R zeolite membranes. **(a)** Scheme of four-channel hollow fiber DD3R zeolite membrane and separation process. **(b)** Fresh membrane, the as-synthesized without any thermal treatment. (**c**) TMCT membrane, 700 °C for 1 min. (**d**) TMCT+CC membrane, 700 °C for 1 min followed by 550 °C for 10 h. (**e**) CC membrane, 550 °C for 10 h with a 0.5 °C min^{-1} heating and cooling rate.

Supplementary Note 1: The color of membrane changed from white (Supplementary Figure 1a) to black (Supplementary Figure 1b), indicating that the template decomposed partly in 1 min at 700 °C. After the following calcination at the temperature of 550 °C, the membrane became light gray, (Supplementary Figure 1c) which was similar with the membrane solely calcined at 550 °C (Supplementary Figure 1d).

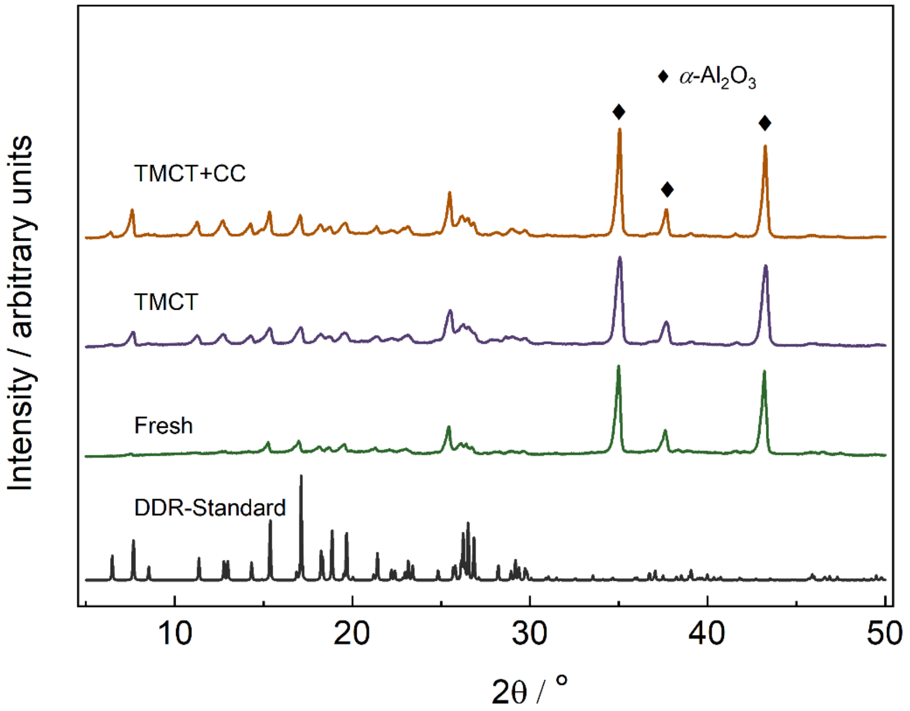

Supplementary Figure 2 PXRD of DD3R zeolite membrane with different stages. Fresh, as-synthesized sample; TMCT, 700 °C for 1 min; TMCT+CC, 700 °C for 1 min followed by 550 °C for 10 h. Source data are provided as a Source Data file.

Supplementary Note 2: As shown in Supplementary Figure 2, the PXRD pattern of fresh membrane matches well with the standard diffraction peaks of DD3R zeolite. The intensities of diffraction peaks especially at low angles are very weak because the membrane is filled with template. After TMCT and the subsequent CC, these peaks turned to be more intensive as the loading amount of template reduced gradually.

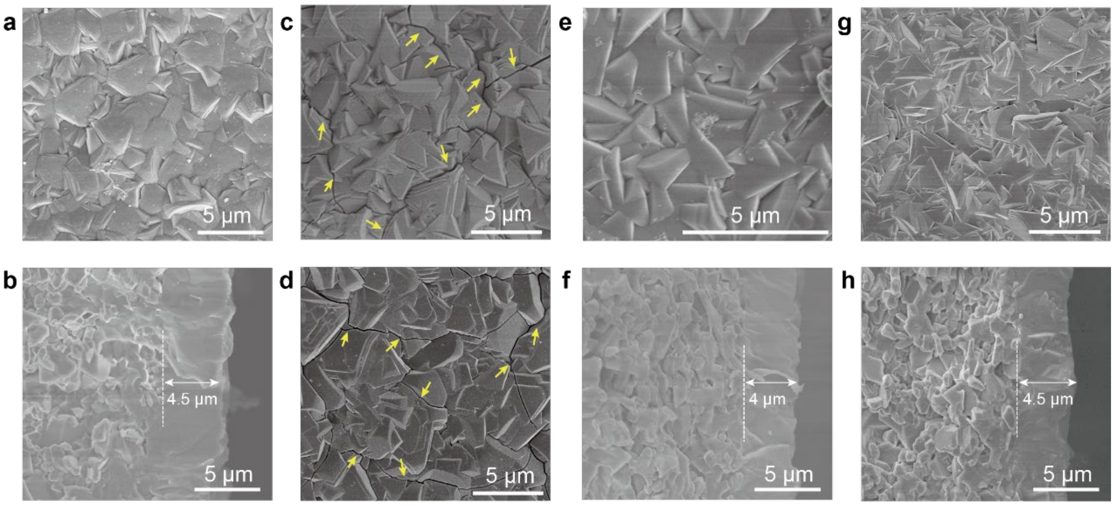

Supplementary Figure 3 Surface and Cross-section SEM images of DD3R zeolite membranes. (**a-b)** Fresh, as-synthesized sample. (**c)** CC700, 700° C for 5 h. (**d)** CC550, 550° C for 10 h. (**e-f)** TMCT, 700 °C for 1 min. (**g-h)** TMCT+CC, 700 °C for 1 min followed by 550 °C for 10 h.

Supplementary Note 3: SEM images in Supplementary Figure 3a and b show that of fresh DD3R zeolite membrane presents a well-intergrown zeolite layer with uniform thickness of ~5 μm on top surface of hollow fiber support.

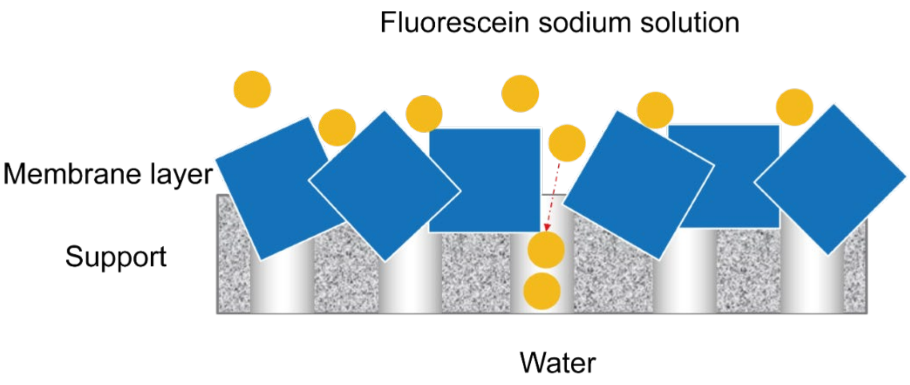

Supplementary Figure 4 Illustration of sample preparation for confocal laser scanning microscopy.

Supplementary Note 4: The hollow fiber zeolite membrane was mounted into a membrane module and the membrane side was contacted with fluorescein sodium solution ($C_{20}H_{10}Na_2O_5$, kinetic diameter~1 nm), while the support side was contacted with DI water (Supplementary Figure 4). The soaking duration is 12 hours at room temperature. Since the kinetic diameter of dye molecule is larger than the zeolitic pore size of DD3R membrane (0.36 nm × 0.44 nm), the dye can only penetrate through and retained in non-crystalline cracks or gaps.

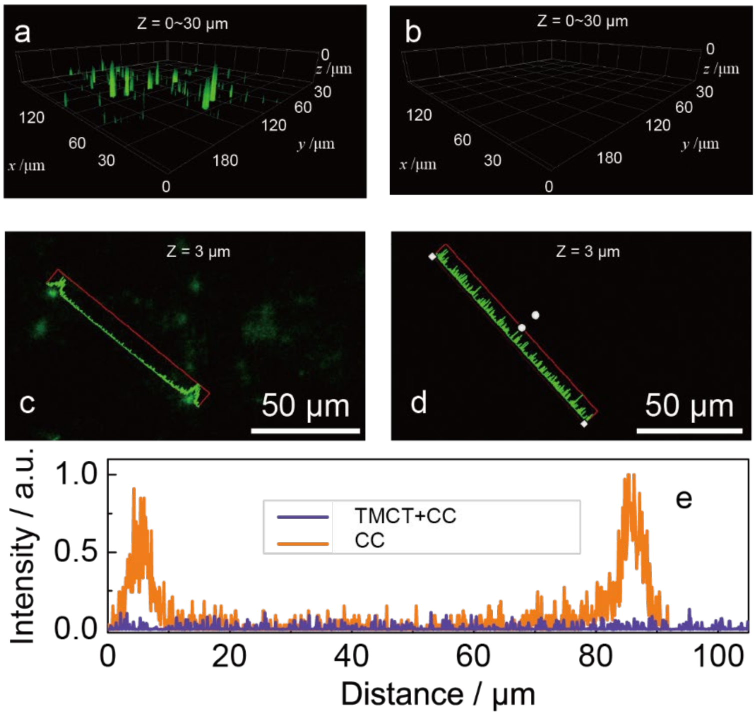

Supplementary Figure 5 Confocal laser scanning microscopy of DD3R membrane. **(a-b)**, 3D visualization of the cracks for the membranes in CC **(a)** and TMCT+CC **(b)** by confocal laser scanning microscopy technique. **(c-e)**, 2D visualization of cracks within the CC **(c)** and TMCT+CC **(d)** membrane layer with a distance of 3 μm to membrane surface. Source data are provided as a Source Data file.

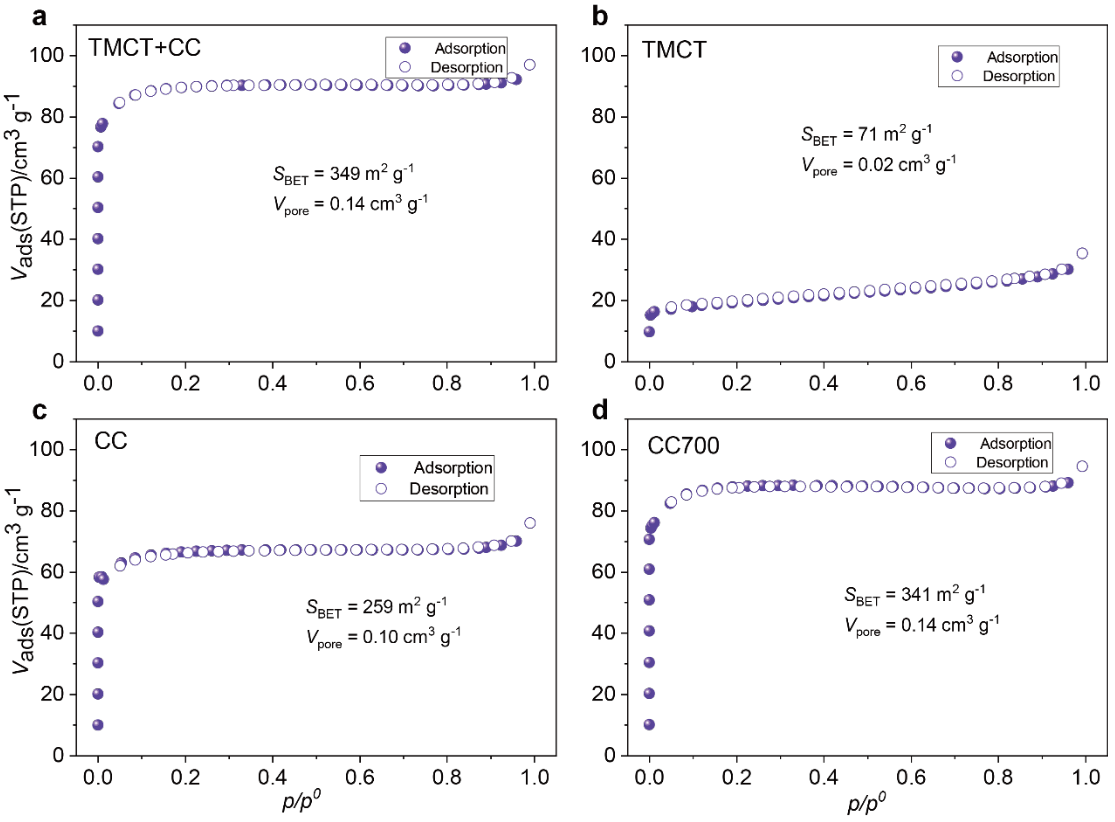

Supplementary Figure 6 N_2 adsorption isotherms. **(a)** TMCT+CC, 700 °C for 1 min followed by 550 °C for 10 h; **(b)** TMCT, 700 °C for 1 min; **(c)** CC, solo 550 °C for 10 h; **(d)** CC700, 700 °C for 5 h. Source data are provided as a Source Data file.

Supplementary Note 5: For all the samples, the N_2 adsorption/desorption isotherms at 77 K are type-I isotherm with no distinct hysteresis loop, which is typical for a microporous material.

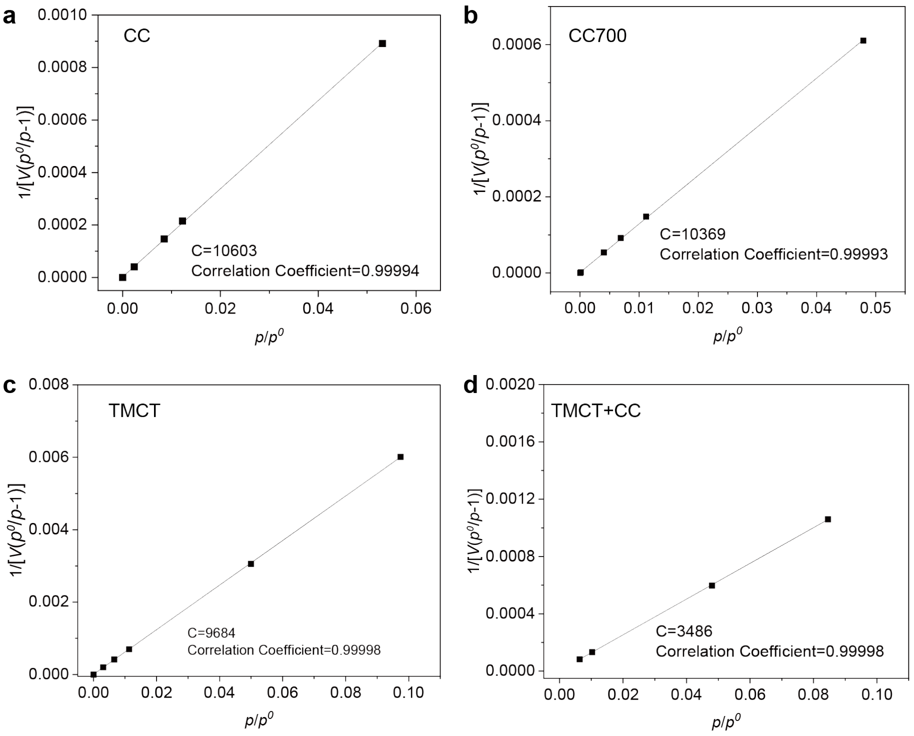

Supplementary Figure 7 BET area calculation process. **(a)** CC, solo 550 °C for 10 h; **(b)** CC700, 700 °C for 5 h; **(c)** TMCT, 700 °C for 1 min; **(d)** TMCT+CC, 700 °C for 1 min followed by 550 °C for 10 h. Source data are provided as a Source Data file.

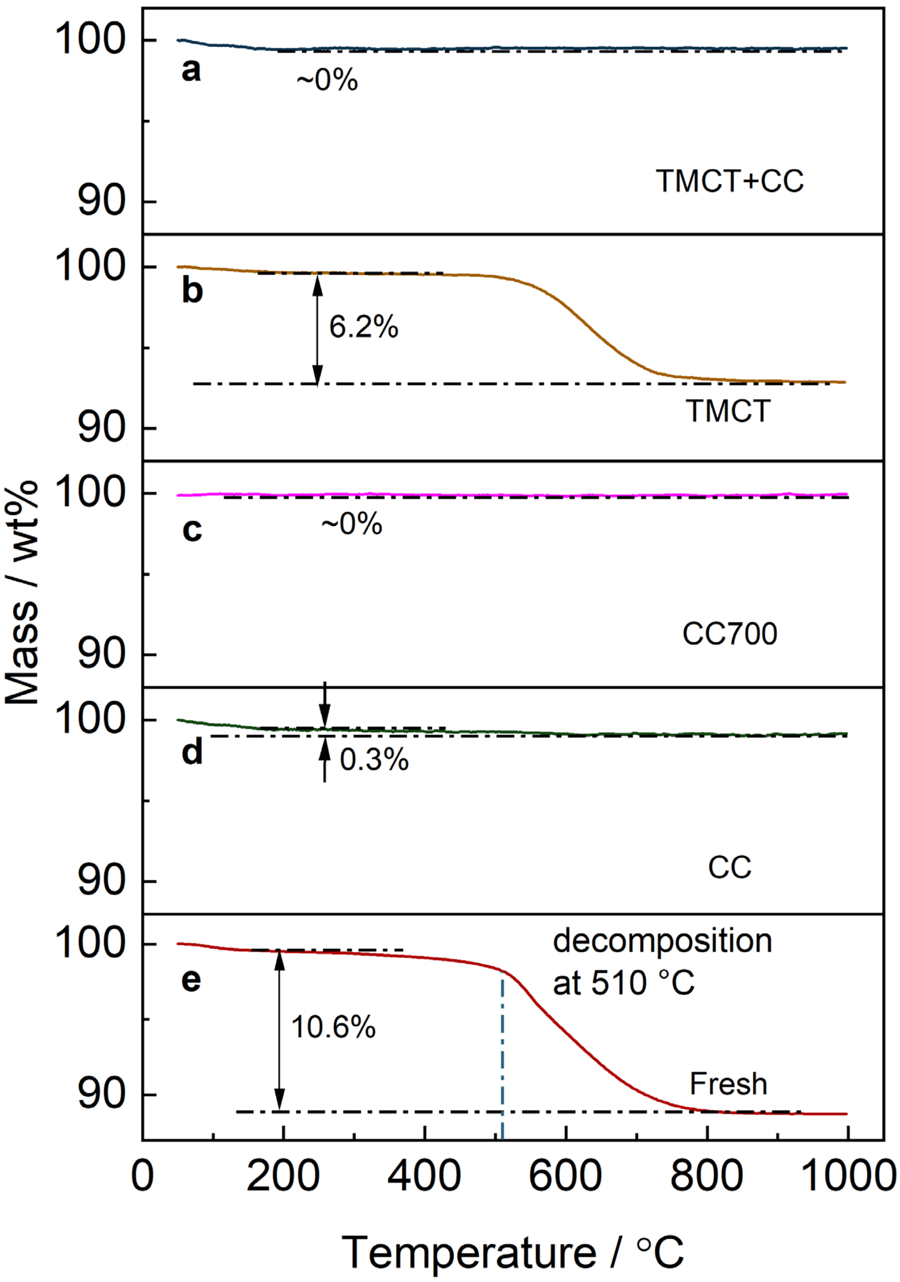

Supplementary Figure 8 TGA results. **(a)** TMCT+CC, 700 °C for 1 min followed by 550 °C for 10 h. (**b)** TMCT, 700 °C for 1 min. (**c)** CC700, 700 °C for 5 h. (**d)** CC, 550 °C for 10 h. **(e)** Fresh, as-synthesized sample. Source data are provided as a Source Data file.

Supplementary Note 6: The degradation of template in DD3R zeolite was also

confirmed by TG results shown in Supplementary Figure 8. The weight loss of all zeolites at below 200 °C is due to desorption of adsorbates (*i.e.* water and CO_2). The fresh DD3R zeolite shows a weight loss of about 10.6 wt% in the temperature range 200-1000 °C, which represents overall removal of template in zeolite, implying the presence of ca. 6 molecules of ADA per unit cell. The weight loss of TMCT-treated zeolite reduced to 6.2 wt%, suggesting that template had cracked partly after the TMCT treatment and the residential accounted for about 43 wt% of the initial template. The weight loss of zeolite treated with TMCT+CC was only less than 0.01 wt%, indicating that OSDAs had been almost removed in the zeolite after the further CC process. Compared to this, the weight loss of zeolite treated with solo CC was a little higher (0.26 wt%), which could be due to incomplete decomposition of OSDAs during the calcination[2].

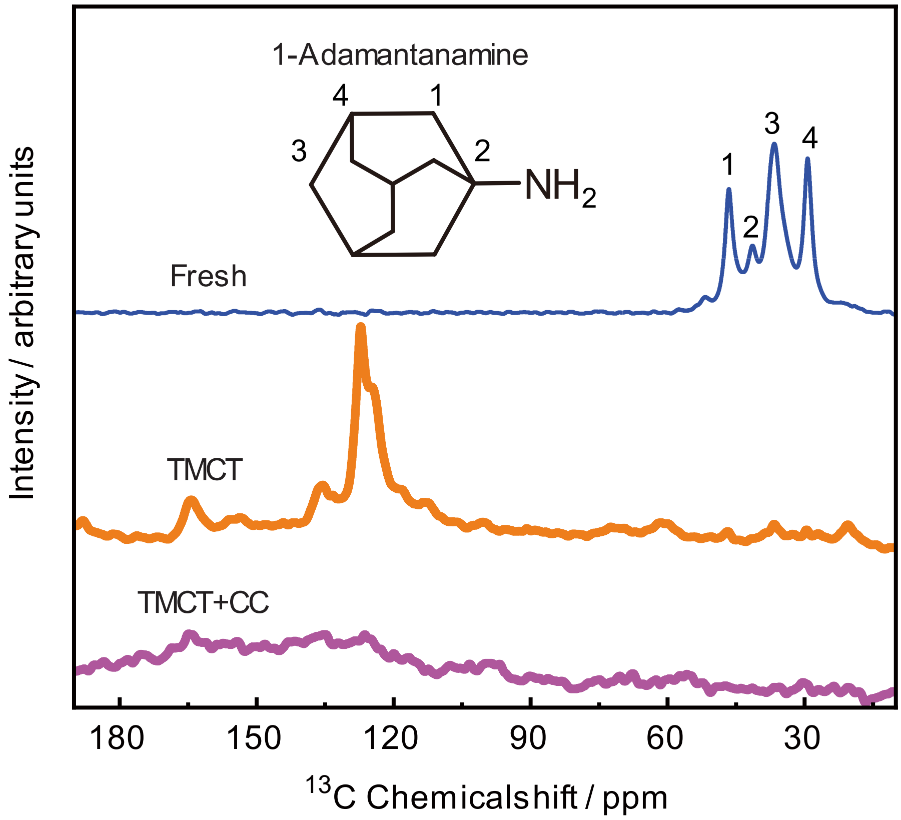

Supplementary Figure 9 ^{13}C NMR spectra of DD3R zeolite with different stages. Fresh, as-synthesized sample; TMCT, 700 °C for 1 min; TMCT+CC, 700 °C for 1 min followed by 550 °C for 10 h. Source data are provided as a Source Data file.

Supplementary Note 7: The structural variation of ADA in DD3R zeolite analyzed by ^{13}C MAS NMR was shown in Supplementary Figure 9 and this figure is same as Fig. 2b. The spectra of as-synthesized DD3R zeolite consist of four characteristic resonances of ADA molecule at 29.4, 36.6, 41.4 and 46.6 ppm. After TMCT, these resonances nearly vanish while some new resonances emerge, implying that the adamantine structure of ADA molecules has cracked partly into

some new products. The small resonance at 20.5 ppm is the characteristic signal of -CH_3. Besides previous analysis, there is another small resonance at 164.2 ppm, which might be some by-product containing carbonyl group. After treated with further CC, there is no strong resonance in the spectra of zeolite, indicating that template has almost decomposed after the calcination.

XPS was further used to analyse the template evolution in zeolitic cavities. The C1*s* spectra of as-synthesized DD3R zeolite was deconvoluted into peaks at 284.8 eV and 286.4 eV, which are assigned to C-C and C-N bonds in 1-adamantanamine molecule (Fig. 2c)[3]. The N 1*s* spectra of fresh zeolite was deconvoluted into two peaks at 399.3 eV and 401.6 eV (Fig. 2d), which could be owed to two different orientations of amino group in 1-adamantanamine molecule in the [$4^3 5^{12} 6^1 8^3$] cage. One type is close to the 6-membered ring and the other type is close to the dodecahedral layer[4].

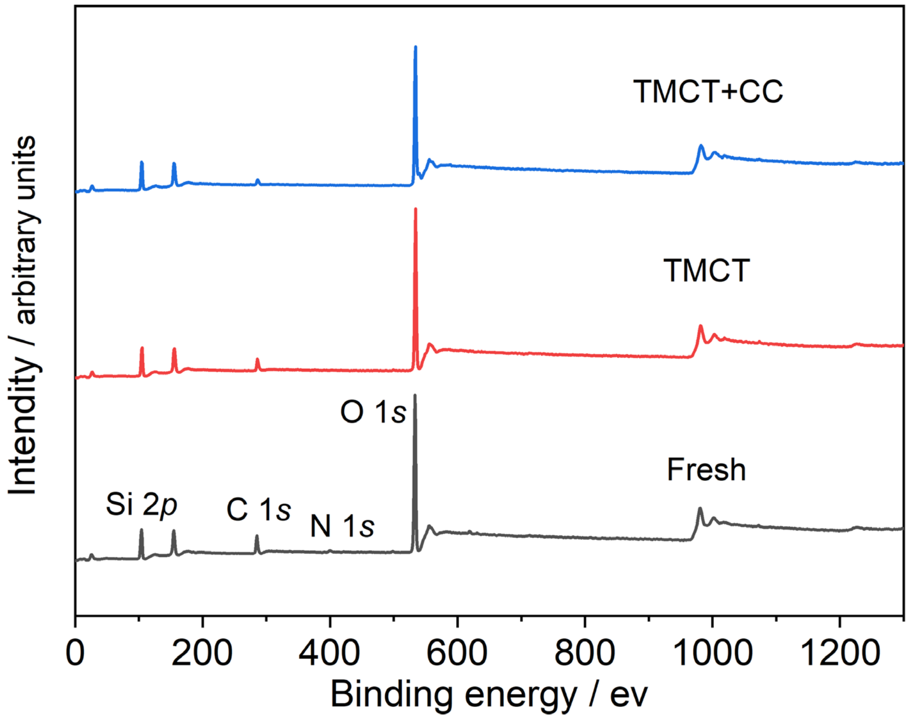

Supplementary Figure 10 The XPS spectrum of DD3R zeolite with different stages. Fresh, as-synthesized sample; TMCT, 700 °C for 1 min; TMCT+CC, 700 °C for 1 min followed by 550 °C for 10 h. Source data are provided as a Source Data file.

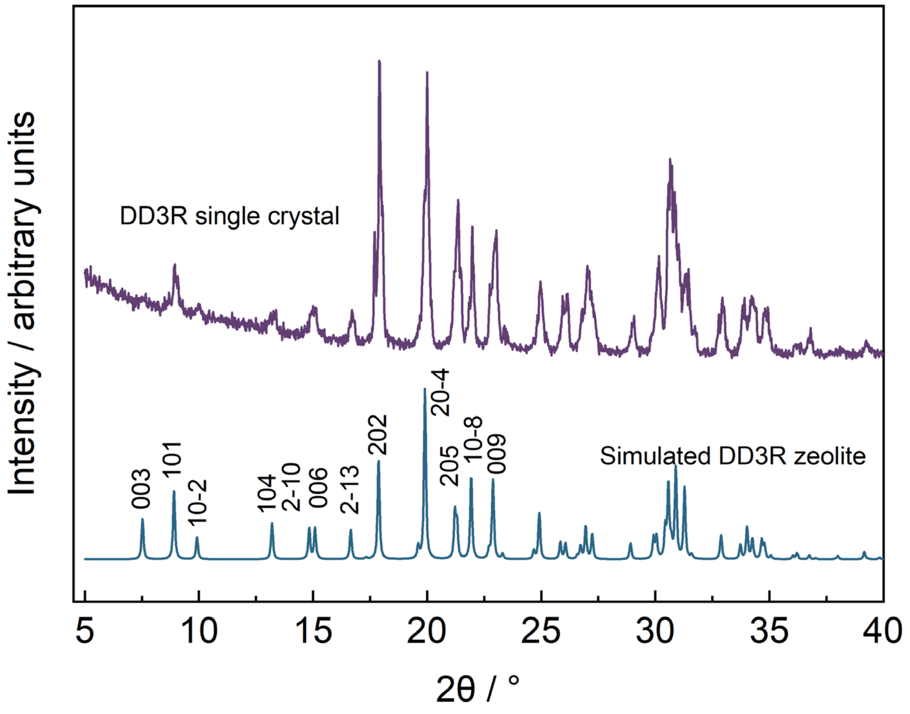

Supplementary Figure 11 PXRD patterns of DDR zeolite crystals. Source data are provided as a Source Data file.

Supplementary Note 8: The XRD pattern of single crystals matches well with the standard diffraction peaks of DDR zeolite.

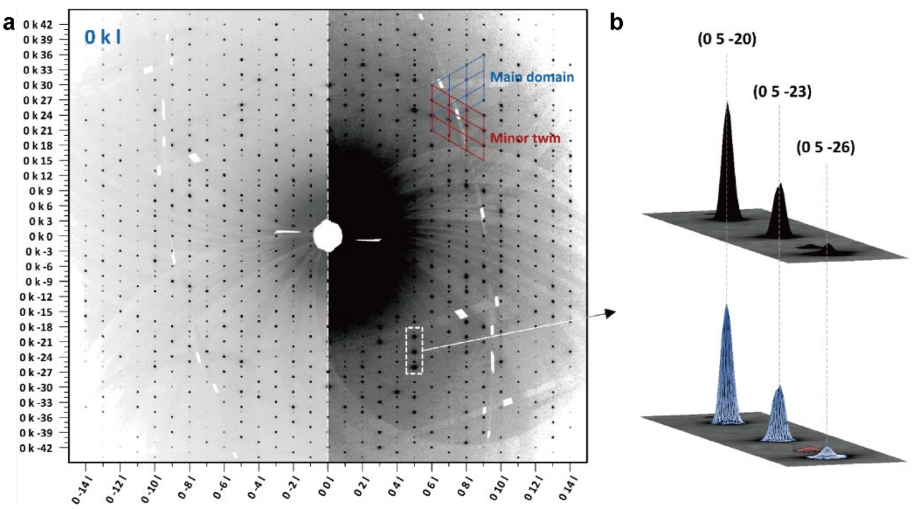

Supplementary Figure 12 (a) reconstruction of the (0 k l) reciprocal lattice plane of the fresh DDR crystal. **(b)** 3D intensity plots of a specific region where is possible to appreciate the presence of diffuse scattering halos centered on the Bragg reflections.

Supplementary Note 9: As shown in Supplementary Figure 12a, the figure is split into two parts to provide a clear picture of the Bragg reflections (left) and of the less intense diffuse scattering (right) by changing the intensity scale. The presence of a minor secondary domain, more evident in the right side of the image, accounts for the presence of additional reflections; two lattices describing the positions of the two domain's reflections in this reciprocal plane are shown in blue and red in the upper-right corner (N.B.: these lattices do not represent the reciprocal lattices of the two domains). The Bragg-to-diffuse intensity ratio is remarkably different comparing the (0 5 -20) and the (0 5 -26) reciprocal lattice positions (Supplementary Figure 12b).

The sample consists of two non-merohedral twin domains. The twin operation is

a 2-fold axes about the (0,0,1) reciprocal lattice vector, with components' fractions 79% vs. 21%. Twin integration resulted in reflection files which did not provide a satisfactory refinement of the crystal phase. Therefore, for structure solution and refinement the data has been integrated considering the main domain only.

A qualitative analysis of the reciprocal space has been carried out based on the precession images calculated from the diffraction data. The sample shows diffuse scattering halos centered on the Bragg reflections, as shown in the following figures. Given the low temperature at which the data collection has been performed (100 K), this diffuse scattering cannot be considered caused by thermal motions of the lattice or to dynamic disorder. Moreover, the Bragg-to-diffuse intensity ratio varies depending on the observed reflection, which further proves that the presence of diffuse intensities is due to the presence of static correlated disorder.

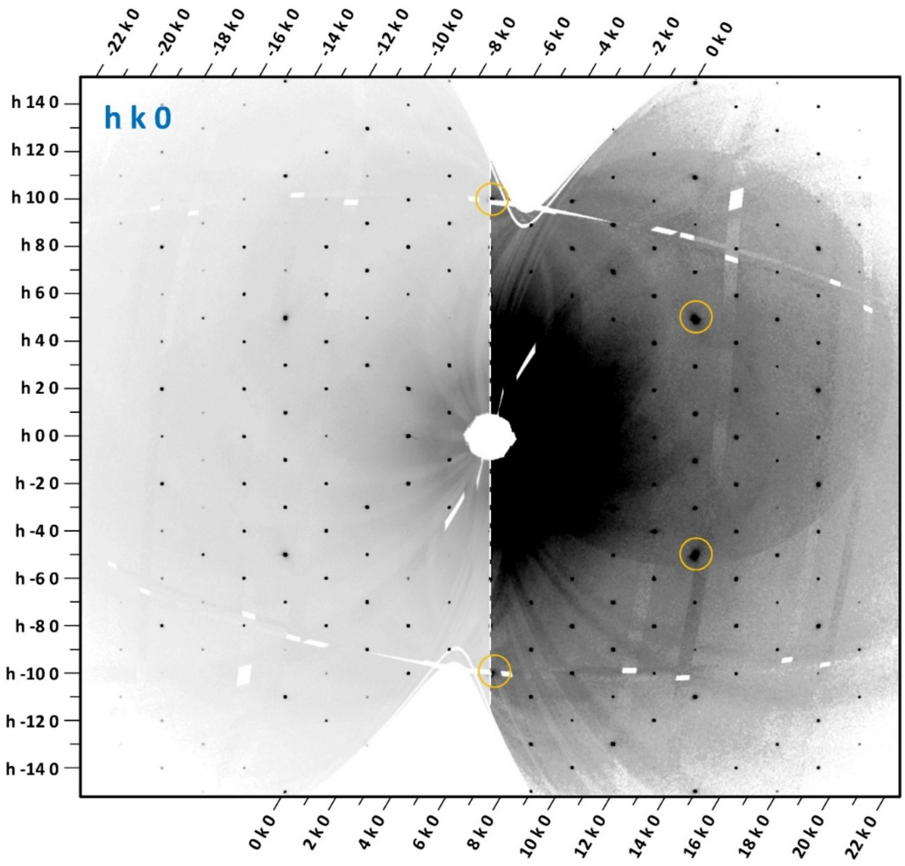

Supplementary Figure 13 Reconstruction of the (h k 0) reciprocal lattice plane of the analyzed fresh DDR crystal.

Supplementary Note 10: The figure is split into two parts to provide a clear picture of the Bragg reflections (left) and of the less intense diffuse scattering (right) by changing the intensity scale. Diffuse scattering halos are visible on reflections (-5 10 0), (5 5 0), (10 -5 0), and (5 -10 0), which are highlighted by yellow circles.

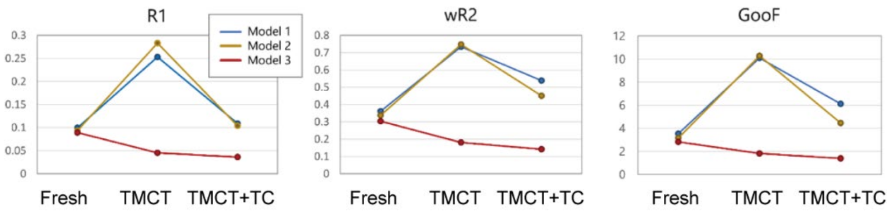

Supplementary Figure 14 Summary of refinement outcomes using different models for the description of the three analyzed states of DDR-type zeolite.

Supplementary Note 11: In order to assess the correct space group for the three presented structures, each one has been described using three different models and the refinements' results were considered to define the most suitable crystallographic model for the analysed samples. All models did not make use of any geometrical restraints, reflections omission or solvent mask. The shelx weighting scheme parameters are set to default values (a = 0.1, b = c = d = e = 0, f = 0.33333). All atoms are refined using anisotropic displacement parameters. The models' different settings are:

Model 1: space group R-3m.

Model 2: space group R-3.

Model 3: space group R-3 and merohedral twinning with twin law [0 1 0, 1 0 0, 0 0 -1].

Crystallographic pictures have been generated using the software Vesta [5] version 3.4.7 and Olex2 [6] version 1.2.10. Reciprocal space reconstructions have been computed

by using the “Unwarping - precession images” feature of the CrysAlisPro software (Rigaku Oxford Diffraction, CrysAlisPro Software System, Version 1.171.38.43, Oxford, UK, 2017) and images have been analysed and exported by using the software CAP FrameView version 1.1 (Copyright © Rigaku Oxford Diffraction 2015). Overall, Model 3 (R-3 space group with merohedral twinning) results the most suitable, although in the case of as-synthesized DDR the differences with respect to the other models are much smaller. The results of this comparative study are reported as follows.

Fresh DDR:

Model 1 refinement outcome: R1 = 0.0997, wR2 = 0.3618, GooF = 3.543

Model 2 refinement outcome: R1 = 0.0935, wR2 = 0.3382, GooF = 3.167

Model 3 refinement outcome: R1 = 0.0893, wR2 = 0.3039, GooF = 2.832, Twin BASF = 0.503(7)

TMCT DDR:

Model 1 refinement outcome: R1 = 0.2532, wR2 = 0.7348, GooF = 10.110

Model 2 refinement outcome: R1 = 0.2834, wR2 = 0.7477, GooF = 10.279

Model 3 refinement outcome: R1 = 0.0456, wR2 = 0.1815, GooF = 1.820, Twin BASF = 0.4954(19)

TMCT+CC DDR:

Model 1 refinement outcome: R1 = 0.1089, wR2 = 0.5388, GooF = 6.119

Model 2 refinement outcome: R1 = 0.1041, wR2 = 0.4509, GooF = 4.455

Model 3 refinement outcome: R1 = 0.0363, wR2 = 0.1424, GooF = 1.398, Twin BASF = 0.4686(18)

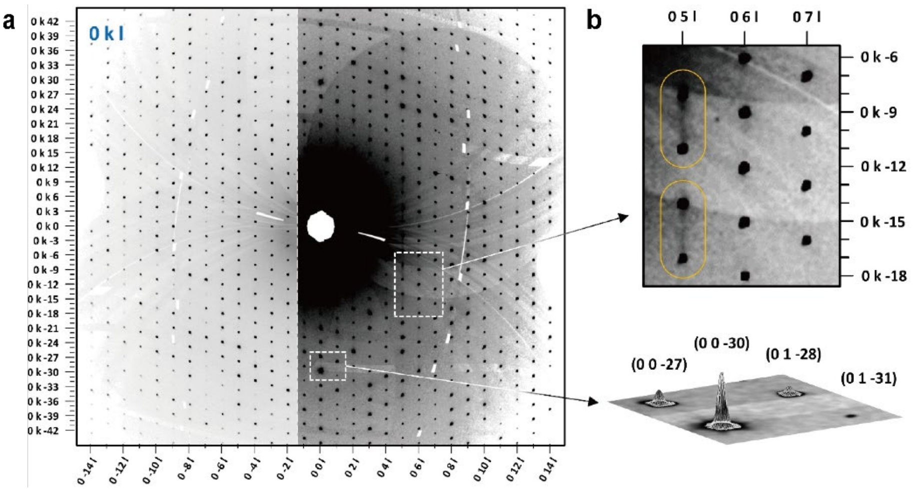

Supplementary Figure 15 (a) Reconstruction of the (0 k l) reciprocal lattice plane of the analyzed TMCT DDR-type crystal. (**b)** magnification of a region where diffuse scattering weak streaks are marked with yellow contours (top) and 3D intensity plot of a selected region where reflections with different Bragg-to-diffuse scattering ratios can be recognized.

Supplementary Note 12: The Supplementary Figure 15a is split into two parts to provide a clear picture of the Bragg reflections (left) and of the less intense diffuse scattering (right) by changing the intensity scale. The sample shows no signs of non-merohedral twinning. Diffuse intensities attributable to correlated disorder can be recognized in the reciprocal space reconstructions shown in the figures below. Overall, the diffuse scattering features is analogous to the one of as-synthesized DDR-type single crystal. However, two features not observed in the previous case are the presence

of diffuse streaks along with c* in the (0 k l) plane and small diffuse clouds localized between specific triads of reflections in the (h k 0) plane (Supplementary Figure 15).

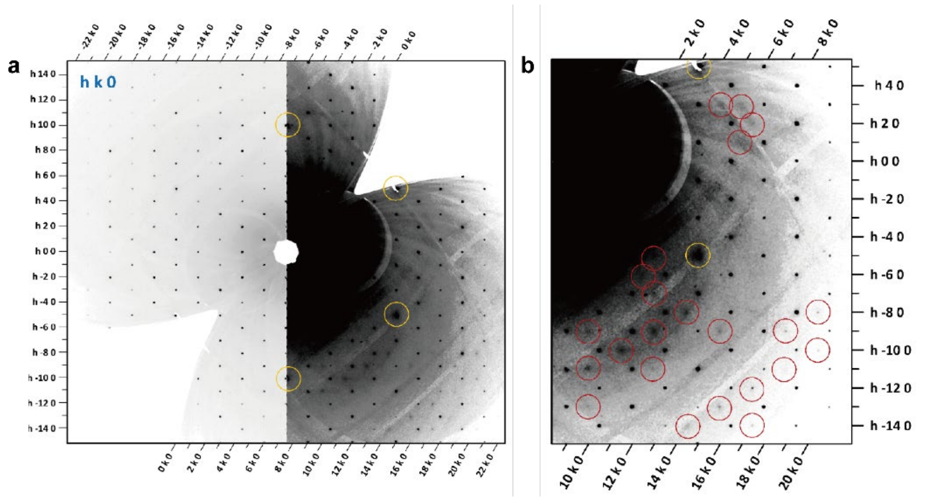

Supplementary Figure 16 (a) Reconstruction of the (h k 0) reciprocal lattice plane of the analyzed TMCT DDR-type crystal. **(b)** other diffuse intensities are localized between certain Bragg peaks' triads, as highlighted with red circles in the magnified picture on the right.

Supplementary Note 13: The Supplementary Figure 16a is split into two parts to provide a clear picture of the Bragg reflections (left) and the less intense diffuse scattering (right) by changing the intensity scale. Diffuse scattering halos are visible on reflections (-5 10 0), (5 5 0), (10 -5 0), and (5 -10 0), which are highlighted by yellow circles.

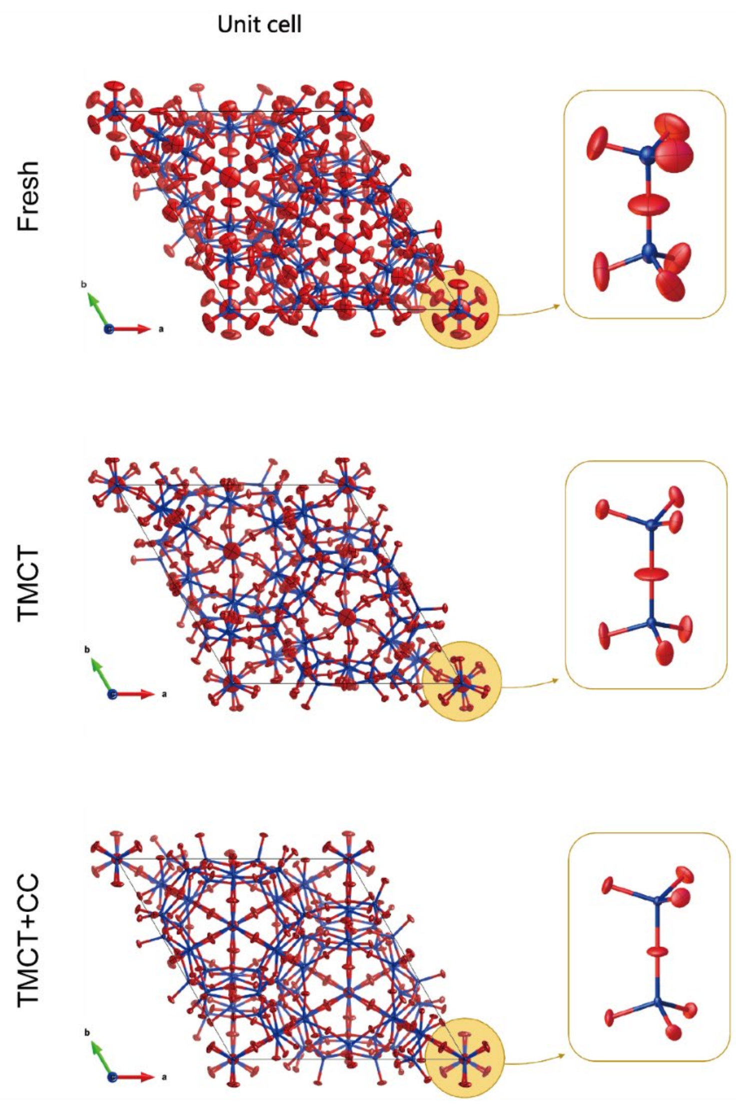

Supplementary Figure 17 Comparison of DDR structures under different stages. Fresh, as-synthesized sample; TMCT, 700 °C for 1 min; TMCT+CC, 700 °C for 1 min followed by 550 °C for 10 h.

Supplementary Note 14: As shown in Supplementary Figure 17, the evident decrease in the Atomic Displacement Parameters (ADPs) of the oxygen atoms in going from the fresh sample to the TMCT one are attributable to a marked decrease in static disorder as consequence of the release of the template molecules from the pores.

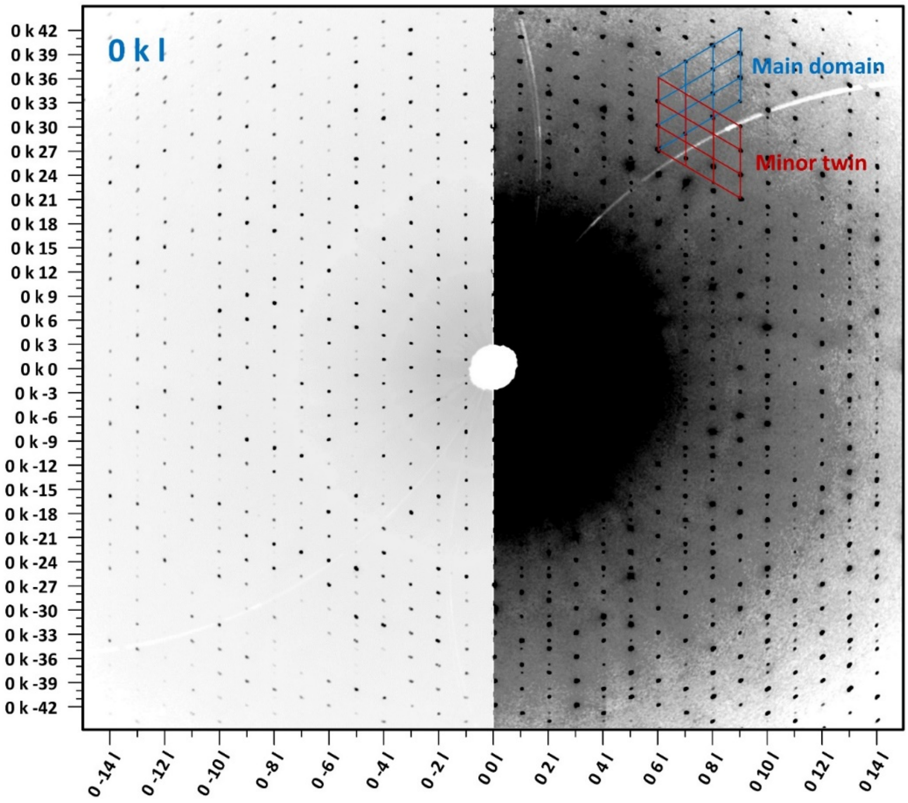

Supplementary Figure 18 Reconstruction of the (0 k l) reciprocal lattice plane of the analyzed TMCT+CC DDR crystal.

Supplementary Note 15: The figure is split into two parts to provide a clear picture of the Bragg reflections (left) and of the less intense diffuse scattering (right) by changing the intensity scale. The features of the diffuse scattering and the additional Bragg intensities due to non-merohedral twinning are analogous to the case of as-synthesized DDR. The sample is affected by non-merohedral twinning of the same type encountered for DDR as-synthesized (twin operation: 2-fold axes about the (0,0,1) reciprocal space vector). The fractions of the two domains are 74% and 26%. As in the case of fresh DDR, also in this case the integration of both domains yielded an

unsatisfactory structure refinement, therefore the only major domain has been used to obtain a refinement with a reliable outcome.

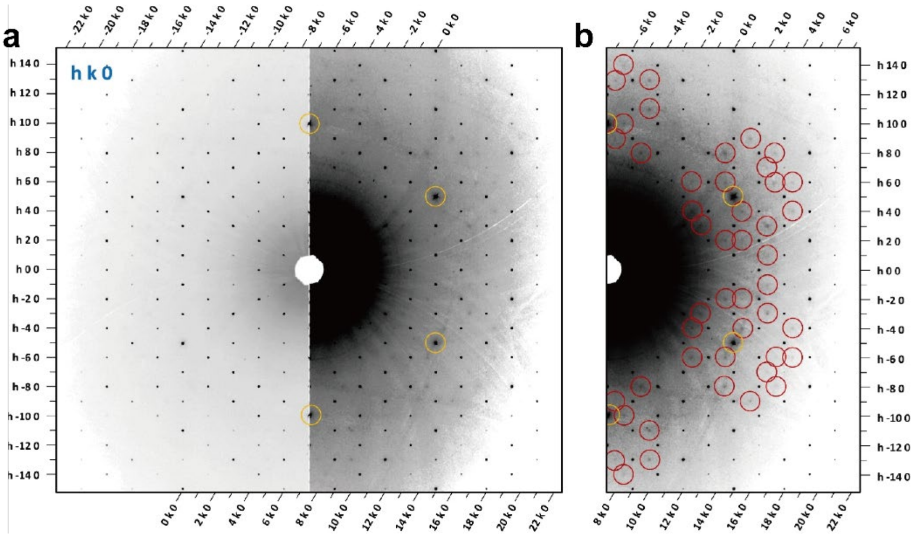

Supplementary Figure 19 (a) Reconstruction of the (h k 0) reciprocal lattice plane of the analyzed TMCT+CC DDR crystal. **(b)** other diffuse intensities are localized between Bragg peaks' triads, as highlighted with red circles in the plot on the right.

Supplementary Note 16: The Supplementary Figure 19a is split into two parts to provide a clear picture of the Bragg reflections (left) and of the less intense diffuse scattering (right) by changing the intensity scale. Diffuse scattering halos are visible on reflections (-5 10 0), (5 5 0), (10 -5 0), and (5 -10 0), which are highlighted by yellow circles.

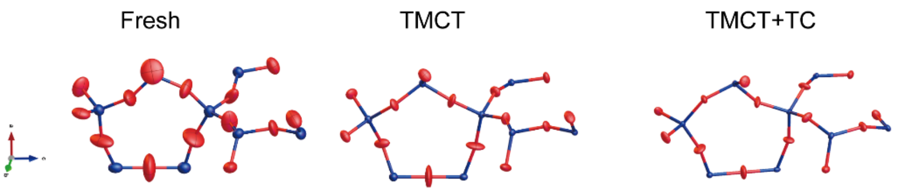

Supplementary Figure 20 Asymmetric unit of DD3R zeolite with different states. Fresh, as-synthesized sample; TMCT, 700 °C for 1 min; TMCT+CC, 700 °C for 1 min followed by 550 °C for 10 h.

Supplementary Note 17: Since the rotation of comer-sharing SiO_4 tetrahedra and ADPs of the oxygen atoms markedly decreasing, 5MR connected with the rotating comer-sharing SiO_4 tetrahedra are bound to change.

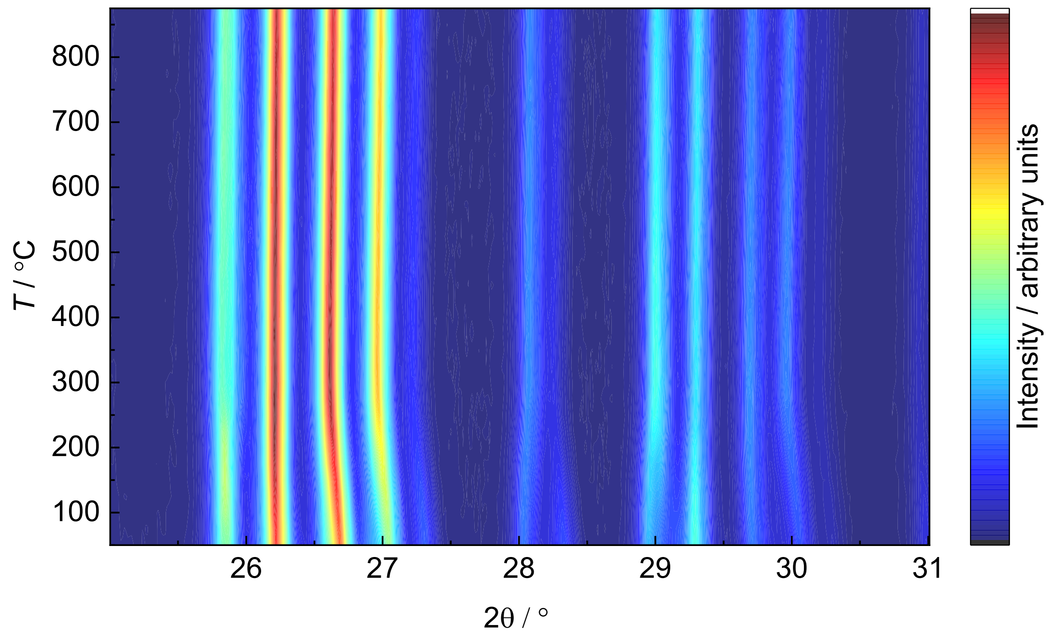

Supplementary Figure 21 Isoline plot of the temperature evolution during in-situ high temperature XRD of empty DD3R zeolite. Source data are provided as a Source Data file.

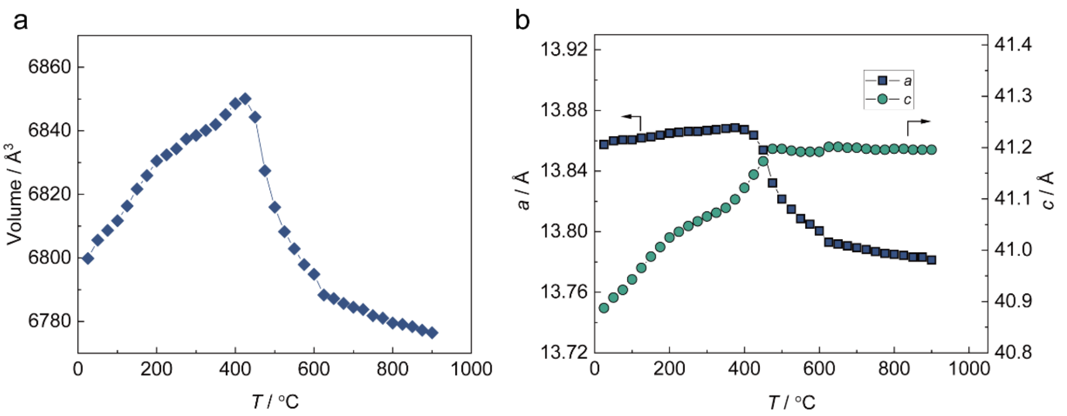

Supplementary Figure 22: Cell parameters and volume evolution as function of temperature for fresh sample. **(a)** cell volume. **(b)** cell parameters. Source data are provided as a Source Data file.

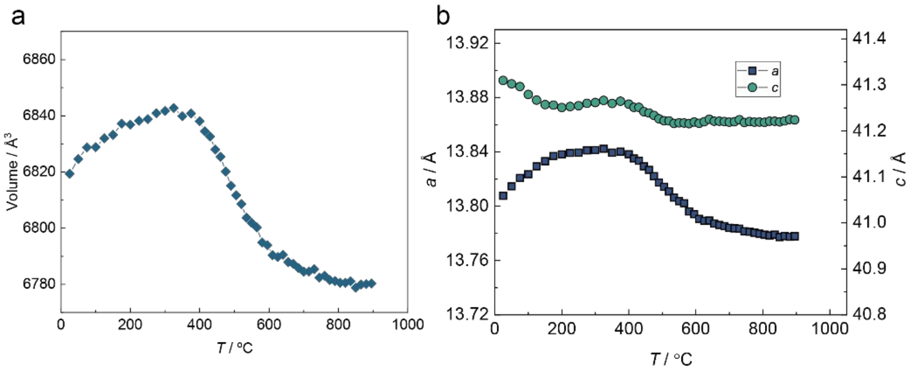

Supplementary Figure 23 Cell parameters and volume evolution as function of temperature for TMCT sample. **(a)** cell volume. **(b)** cell parameters. Source data are provided as a Source Data file.

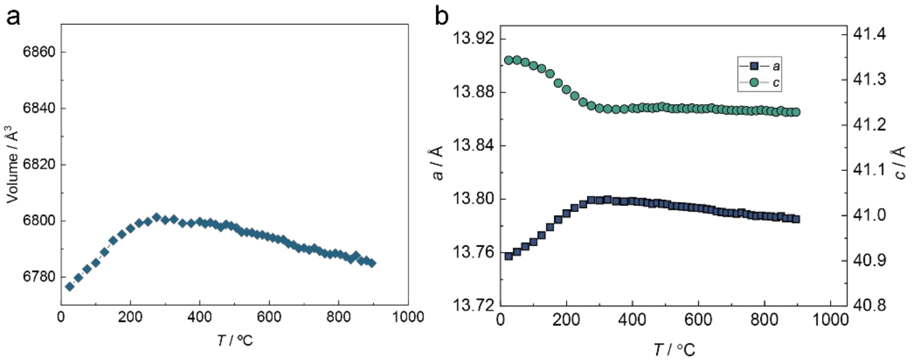

Supplementary Figure 24 Cell parameters and volume evolution as function of temperature for empty sample. **(a)** cell volume. **(b)** cell parameters. Source data are provided as a Source Data file.

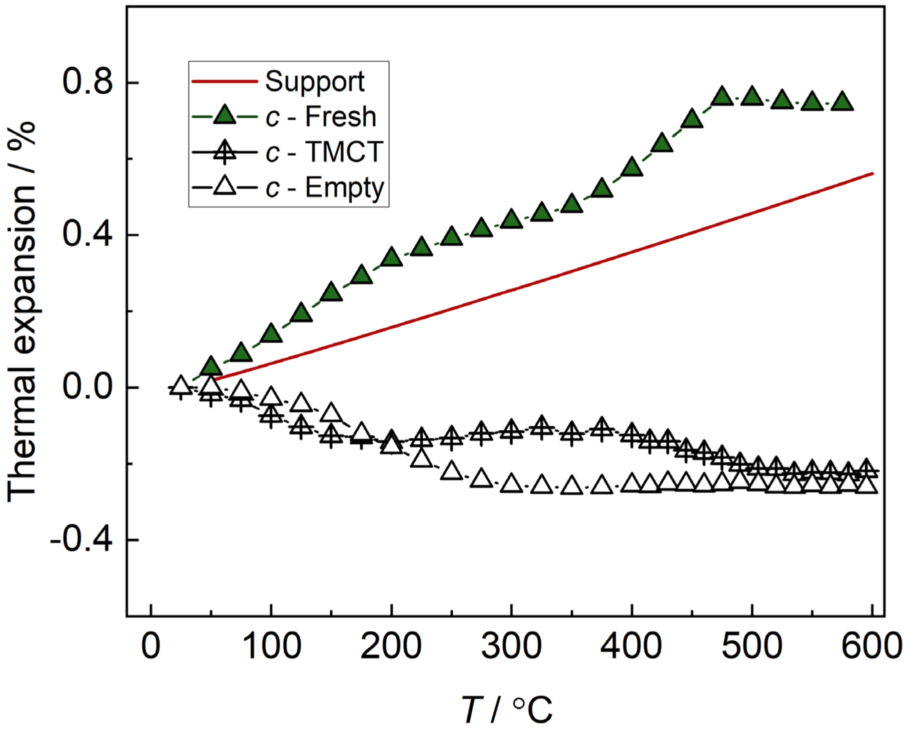

Supplementary Figure 25 Thermal expansion of Al_2O_3 support (relative change in length) and *c* parameter. Source data are provided as a Source Data file.

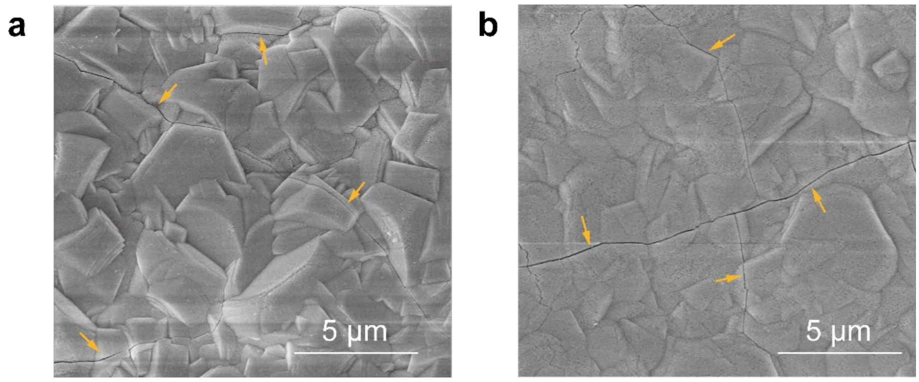

Supplementary Figure 26 SEM images of DD3R zeolite membranes with different TMCT periods: **(a)** 8 min. (**b)** 60 min.

Supplementary Note 18: Defects are clearly seen in the membranes treated with TMCT for 8 and 60 min, indicating that the periods of TMCT treatment at 700 °C were over long.

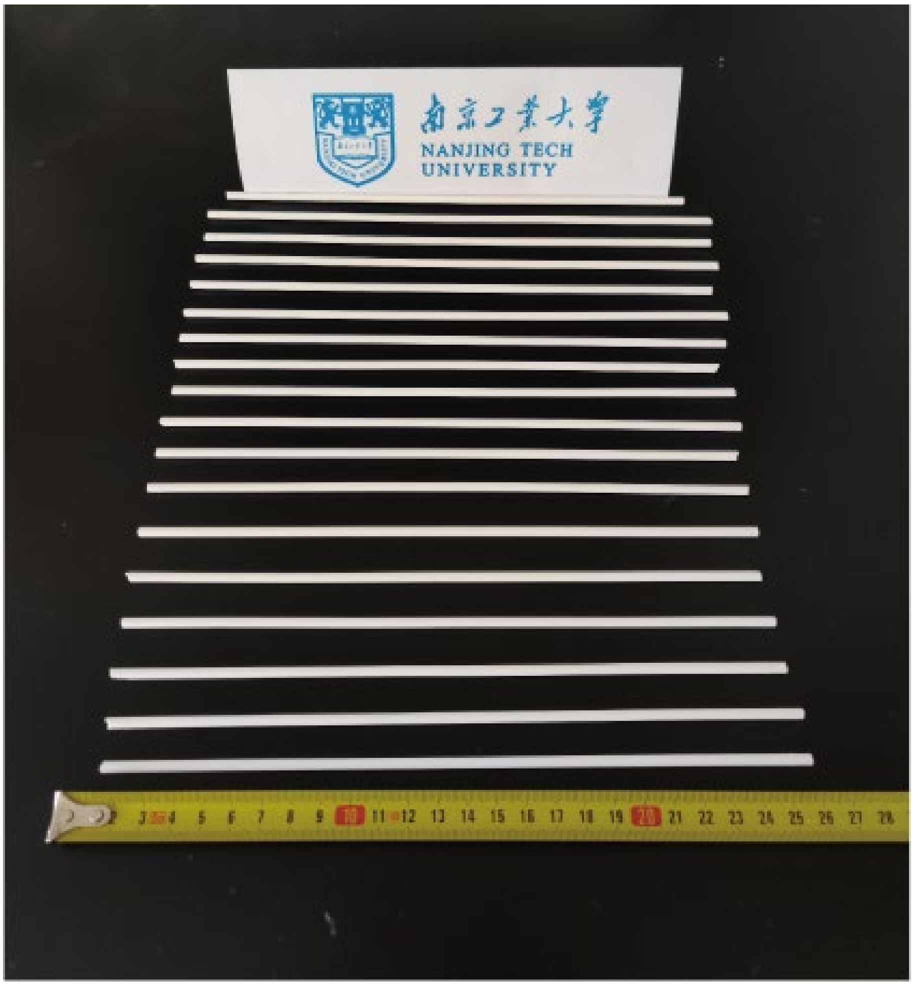

Supplementary Figure 27 Picture of 20 cm long hollow fiber supported DD3R zeolite membranes.

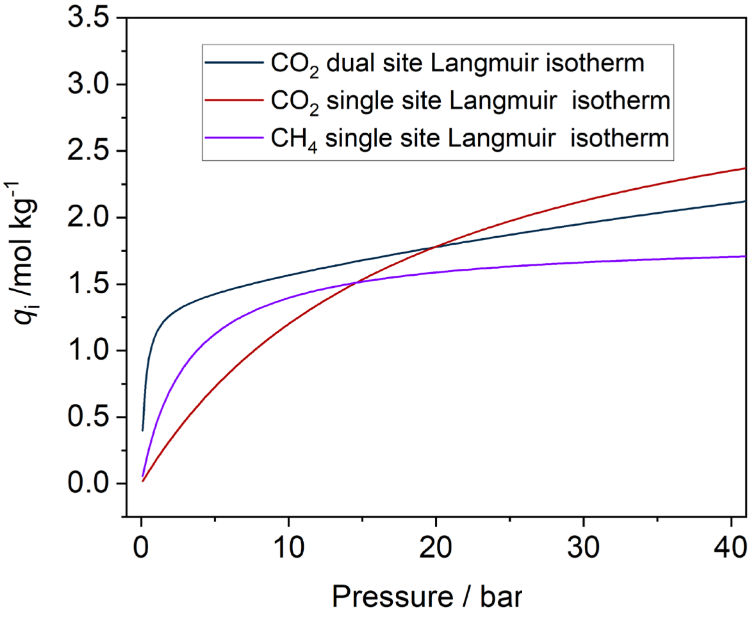

Supplementary Figure 28 Simulated adsorption isotherms of CO_2 and CH_4 in DD3R zeolite. Source data are provided as a Source Data file.

Supplementary Note 19: The adsorption isotherms of CO_2 were simulated by a single (Eq. (2)) and dual site Langmuir model (Eq. (3)). The adsorption isotherm of CH_4 was simulated by a single (Eq. (2)) site Langmuir model. The parameters of model were from literature[7, 8].

$$q_i = q_i^{sat,A} \frac{K_i^A p_i}{1+K_i^A p_i}, K_i^n = K_0^n e^{\frac{-\Delta H_I^n}{RT}} \tag{2}$$

$$q_i = q_i^{sat,A} \frac{K_i^A p_i}{1+K_i^A p_i} + q_i^{sat,B} \frac{K_i^B p_i}{1+K_i^B p_i}, K_i^n = K_0^n e^{\frac{-\Delta H_I^n}{RT}} \tag{3}$$

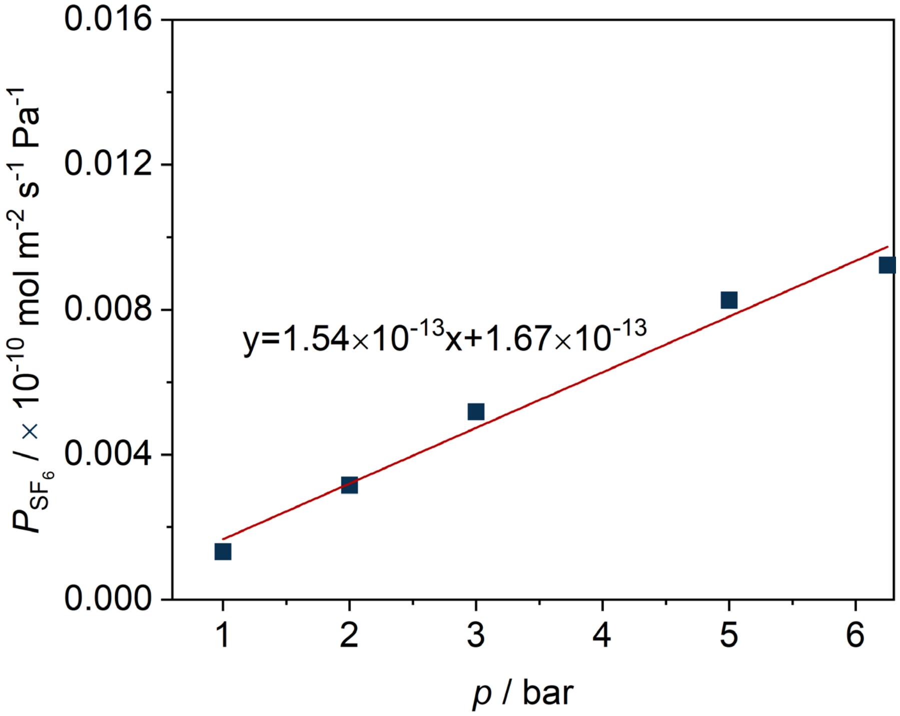

Supplementary Figure 29 SF_6 single gas permeance as function of pressure drop. Source data are provided as a Source Data file.

Supplementary Note 20: SF_6 permeance matched well with linear rising trend with the pressure drop. It can be calculated that SF_6 permeance consists of two parts: the independent Knudsen permeance of 1.67×10^{-13} mol m^{-2} s^{-1} Pa^{-1} and linearly increased viscous flow permeance dependent on pressure drop.

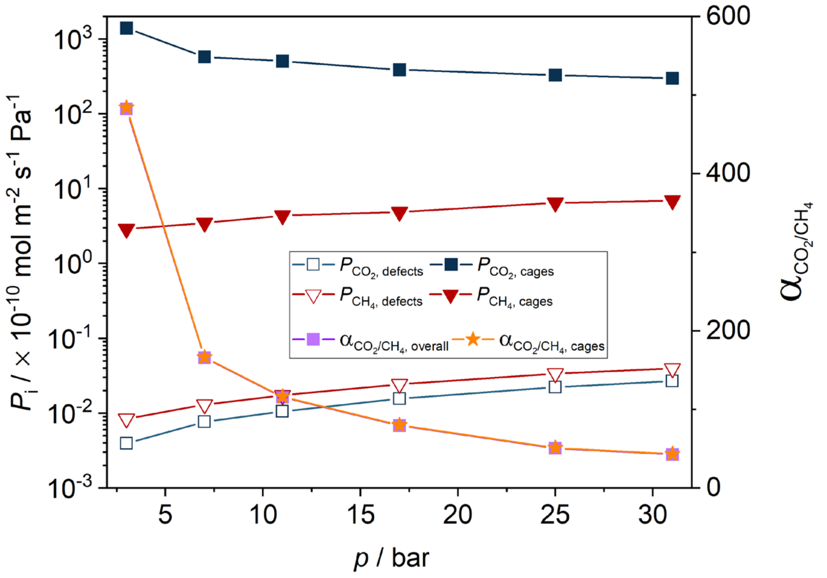

Supplementary Figure 30 Equimolar CO_2 and CH_4 binary gas separation performance as a function of feed pressure. Effective membrane length: 20 cm. Source data are provided as a Source Data file.

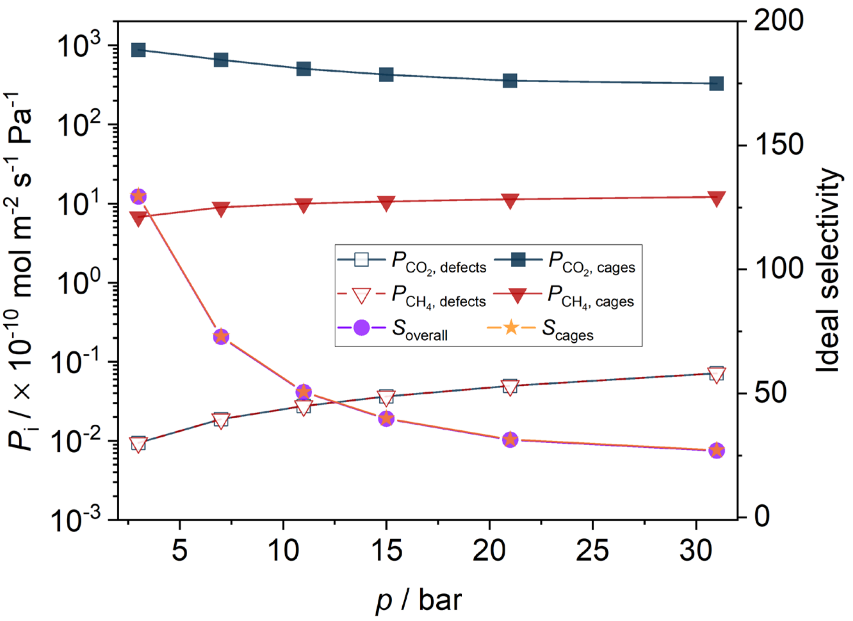

Supplementary Figure 31 CO_2 and CH_4 single gas permeance and ideal selectivity as a function of feed pressure. Effective membrane length: 20 cm. Source data are provided as a Source Data file.

Supplementary Note 21: The slight decrease of CO_2 permeance through DD3R cages is attributed to the monotonic decrease of CO_2 diffusivity at higher loading by nonlinear adsorption[9, 10]. On the contrary, CH_4 permeance contributed by DD3R cages slightly increased with pressure due to the improved diffusivity[10]. Both CO_2 and CH_4 permeance through the defects increased with pressure because of the presence of viscous flow. The overall CO_2 and CH_4 permeance were shown in Supplementary Table 5.

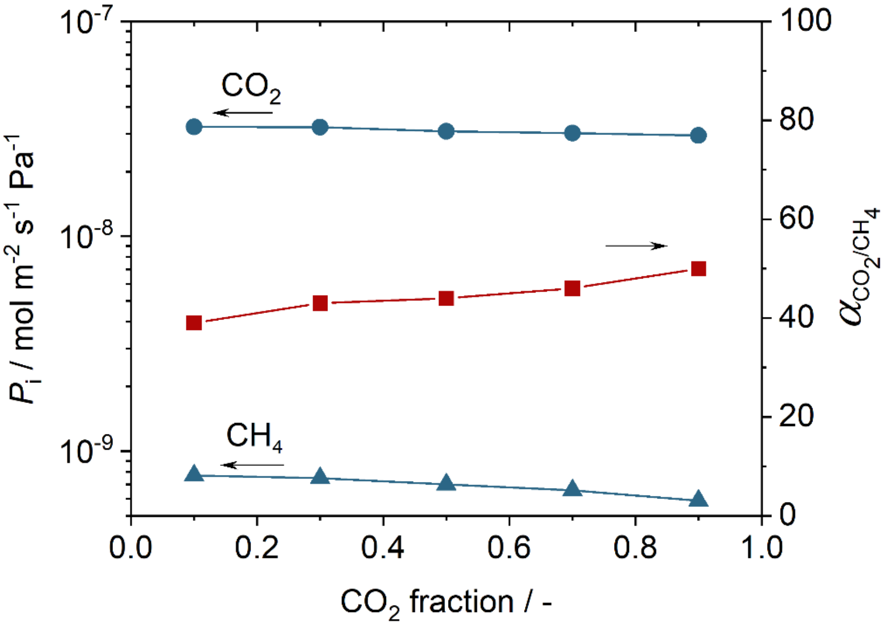

Supplementary Figure 32 Effect of molar fraction on CO_2/CH_4 mixture separation at 31 bar. No sweep gas. Source data are provided as a Source Data file.

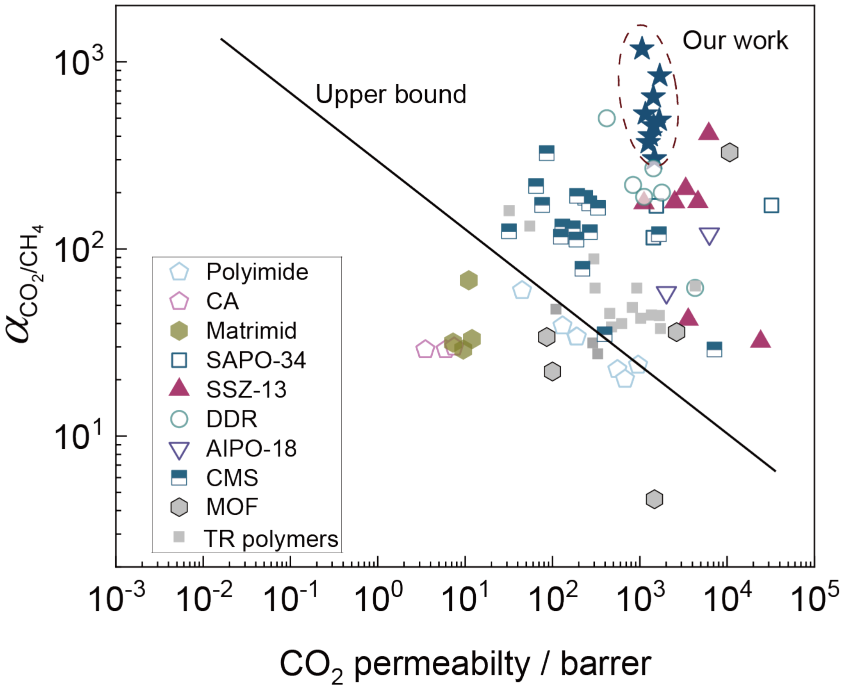

Supplementary Figure 33 Comparison on CO_2/CH_4 separation performance, involving commercial cellulose acetate (CA) membranes[11], polyimide membranes[12], Matrimid membranes[13], carbon molecular sieve (CMS) membranes[14, 15, 16], MOF membranes[17, 18, 19] and other 8MR zeolite membranes [11, 12, 13, 20, 21, 22, 23, 24, 25, 26, 27, 28, 29, 30, 31, 32, 33, 34, 35, 36, 37, 38, 39, 40, 41, 42]. Source data are provided as a Source Data file. The image was shown as Fig. 5c in the main text.

Supplementary Table 1 The crystallographic table of DDR-type single crystals.

	Fresh	TMCT	TMCT+CC
Empirical formula	O_2Si	O_2Si	O_2Si
Formula weight	60.09	60.09	60.09
Temperature/K	100	100	100
Crystal system	trigonal	trigonal	trigonal
Space group	*R-3m*	*R-3*	*R-3*
a/Å	13.77360(10)	13.71776(8)	13.80320(10)
b/Å	13.77360(10)	13.71776(8)	13.80320(10)
c/Å	40.7783(2)	41.29196(19)	40.7853(2)
α/°	90	90	90
β/°	90	90	90
γ/°	120	120	120
Volume/$Å^3$	6699.69(10)	6729.18(8)	6729.67(10)
Z	124	119.99988	120
$\rho_{calc}g/cm^3$	1.817	1.779	1.779
μ/mm^{-1}	0.458	0.453	0.453
F (000)	3660.0	3600.0	3600.0
Crystal size/mm^3	0.07 × 0.07 × 0.07	0.1 × 0.1 × 0.1	0.08 × 0.08 × 0.08
Radiation	synchrotron (λ = 0.6199)	synchrotron (λ = 0.6199)	synchrotron (λ = 0.6199)

2θ range for data collection/°	3.102 to 56.956	3.45 to 56.954	3.096 to 56.94
Index ranges	-19 ≤ h ≤ 20, -21 ≤ k ≤ 21, -62 ≤ l ≤ 62	-19 ≤ h ≤ 19, -21 ≤ k ≤ 21, -63 ≤ l ≤ 63	-21 ≤ h ≤ 21, -21 ≤ k ≤ 20, -62 ≤ l ≤ 62
Reflections collected	53754	53358	55377
Independent reflections	3143 [R_{int} = 0.0540, R_{sigma} = 0.0149]	5680 [R_{int} = 0.0390, R_{sigma} = 0.0142]	5675 [R_{int} = 0.0619, R_{sigma} = 0.0213]
Data/restraints/parameters	3143/0/163	5680/0/183	5675/0/183
Goodness-of-fit on F^2	1.085	1.143	1.078
Final *R* indexes [I>=2σ (I)]	R_1 = 0.0611 wR_2 = 0.1834	R_1 = 0.0376 wR_2 = 0.1040	R_1 = 0.0277 wR_2 = 0.0777
Final *R* indexes [all data]	R_1 = 0.0617 wR_2 = 0.1839	R_1 = 0.0376 wR_2 = 0.1040	R_1 = 0.0277 wR_2 = 0.0777
Largest diff. peak/hole / e Å^{-3}	1.27/-0.90	1.19/-0.84	1.21/-0.60

Supplementary Table 2 Thermal expansion coefficients of different zeolites.

Framework	Stage	Temp. range / °C	Thermal expansion coefficient / $°C^{-1}$	Ref.
DD3R	Fresh powder	25-425	1.87×10^{-5}	This work
	TMCT powder	25-325	1.14×10^{-5}	
	Empty powder	25-275	1.46×10^{-5}	
		275-895	-3.87×10^{-6}	
DD3R	Empty powder	<300	1.8×10^{-5}	[43]
	Empty single crystal	<227	1.4×10^{-5}	
	Empty powder	>300	-3×10^{-6}	
DD3R	Empty powder	25-219	3.51×10^{-5}	[44]
	Empty powder	219-912	-8.7×10^{-6}	
MFI	Empty powder	25-75	2.7×10^{-5}	[44]
		120-702	-1.51×10^{-5}	
CHA	Empty powder	20-600	-2.85×10^{-5}	[45]

Supplementary Table 3 CO_2/CH_4 separation performance of the 20 cm long DD3R zeolite membranes [a].

Number	P_{CO2} [b]	$\alpha_{CO2/CH4}$	Number	P_{CO2} [b]	$\alpha_{CO2/CH4}$
1	1,400	487	10	1,230	303
2	1,100	403	11	870	300
3	1,000	211	12	1,200	650
4	1,080	326	13	950	204
5	1,210	455	14	970	277
6	1,050	370	15	1,540	157
7	970	527	16	1,150	174
8	890	1172	17	1,400	320
9	1,420	843			

[a]: pressure of 1 bar at 25 °C, 50/50 CO_2/CH_4 mixture, sweep gas He flow rate: 100 mL min^{-1}; [b]: $\times 10^{-10}$ mol m^{-2} s^{-1} Pa^{-1}

Supplementary Table 4 Separation performance of MFI and SSZ-13 zeolite membranes detemplated by convention calcination (CC) and TMCT method.

No	Membrane	Detemplation	Separation performance		
			Feed	P_i / mol m^{-2} s^{-1} Pa^{-1}	α
M1	MFI	CC450 [c]	PX/OX [a]	2.6×10^{-8}	11
M2	MFI	CC450 [c]	PX/OX [a]	2.1×10^{-8}	20
M3	MFI	TMCT [d]	PX/OX [a]	2.6×10^{-8}	82
M4	MFI	TMCT [d]	PX/OX [a]	4.7×10^{-8}	45
M5	SSZ-13	CC450 [c]	CO_2/CH_4 [b]	2.4×10^{-7}	77
M6	SSZ-13	CC450 [c]	CO_2/CH_4 [b]	2.6×10^{-7}	81
M7	SSZ-13	TMCT [d]	CO_2/CH_4 [b]	3.4×10^{-8}	11
		TMCT [d] +CC450 [c]	CO_2/CH_4 [b]	2.0×10^{-7}	41
M8	SSZ-13	TMCT [d]	CO_2/CH_4 [b]	5.1×10^{-8}	22
		TMCT [d] +CC450 [c]	CO_2/CH_4 [b]	2.4×10^{-7}	100

[a]: both components had same partial pressure of 2 kPa at 150 °C; [b]: equimolar CO_2/CH_4 binary mixture at 25 °C and feed pressure of 2 bar; [c]: calcination at 450 °C for 12 h with heating and cooling rate of 0.5 °C min^{-1}; [d]: TMCT at 700 °C for 1 min.

Supplementary Note 22: Here we would like to share some preliminary results of MFI and SSZ-13 zeolite membranes (Supplementary Table 4). (1) MFI zeolite membranes: Two MFI zeolite membranes were detemplated with conventional calcination (CC) and TMCT method, respectively. In the case of CC-treated membranes (M1 and M2), the selectivity of *p*-xylene (PX) over *o*-xylene (OX) were 11 and 20. However, the selectivity was up to 45 and 82 if TMCT method was adopted (M3 and

M4). This would be another evidence to our proposal that the diffusion resistance of decomposed species is one of key points for detemplation. MFI zeolite has larger effective pore size (0.55 × 0.51 nm and 0.56 × 0.53 nm) than DD3R zeolite (0.36 × 0.44 nm). Its template (tetrapropylammonium cations, TPA^+) can be easily removed by sole TMCT treatment at 700 °C for 1 minute. In that case, the PX permeance of TMCT-treated membrane was comparable to that of the CC-treated one. (2) SSZ-13 zeolite membranes: Similar experiment was conducted on SSZ-13 zeolite membranes (M7 and M8, Supplementary Table 4). After TMCT treatment, the membranes showed CO_2/CH_4 selectivity of 11 and 22, while the CO_2 permeance was 3.4×10^{-8} mol m^{-2} s^{-1} Pa^{-1} and 5.1×10^{-8} mol m^{-2} s^{-1} Pa^{-1}. Interestingly, the CO_2 permeance increased 5-fold and reached 2.4×10^{-7} mol m^{-2} s^{-1} Pa^{-1} after the following CC treatment. Simultaneously, the CO_2/CH_4 selectivity increased up to 100 even higher than the conventional calcinated membranes (M5 and M6). The results matched well with DD3R zeolite membranes as demonstrated in the Revised Manuscript. Therefore, the TMCT approach was universally efficient to other zeolite membranes (*e.g.*, MFI and SSZ-13).

Supplementary Table 5 Contribution of surface diffusion (zeolitic cages), Knudsen diffusion and viscous flow of CO_2 and CH_4 permeation through DD3R zeolite membranes in Fig 5d.

Pressure/ bar	CO_2 (single gas)[b]				CH_4 (single gas) [b]				CO_2 (binary mixture) [b]				CH_4 (binary mixture) [b]			
	$P^{a}_{Measured}$	P^{a}_{Vis}	P^{a}_{Kn}	f^{e}_{defect}	$P^{a}_{Measured}$	P^{a}_{Vis}	P^{a}_{Kn}	f^{e}_{defect}	$P^{a}_{Measured}$	P^{a}_{Vis}	P^{a}_{Kn}	f^{e}_{defect}	$P^{a}_{Measured}$	P^{a}_{Vis}	P^{a}_{Kn}	f^{e}_{defect}
3	878	0.0033	0.00305	0.0007	6.8	0.0044	0.0051	0.14	1400	0.0009	0.0031	0.0003	2.9	0.0034	0.0051	0.2899
7	655	0.0098	0.00305	0.002	9.0	0.0132	0.0051	0.21	580	0.0043	0.0031	0.0013	3.5	0.0079	0.0051	0.3711
1.1	506	0.0163	0.00305	0.004	10.0	0.0219	0.0051	0.28	510	0.0071	0.0031	0.0021	4.4	0.0123	0.0051	0.3947
1.5	425	0.0228	0.00305	0.006	10.7	0.0307	0.0051	0.34	390	0.0122	0.0031	0.0040	4.9	0.0194	0.0051	0.4987
21	357	0.0325	0.00305	0.010	11.4	0.0439	0.0051	0.44	330	0.0188	0.0031	0.0067	6.5	0.0289	0.0051	0.5221
31	329	0.0488	0.00305	0.016	12.2	0.0658	0.0051	0.59	300	0.0235	0.0031	0.0090	7.0	0.0346	0.0051	0.5659

[a] : $\times 10^{-10}$ mol m^{-2} s^{-1} Pa^{-1}; [b]: the dynamic viscosity is 15.123 μPa s for SF_6, 14.687 μPa s for CO_2, 17.9 μPa s for CH_4; [e] : Fraction of permeance through defects, %. A rigid DD3R zeolite framework was assumed here.

Supplementary Table 6 Comparison in separation performance between 8MR all-silica or high-silica zeolite membranes.

Type	Synthesis method	Support	$S_{membrane}$ / cm^2	Template removal	Thickness/μm	P / bar [a]	P_{CO2} [a],	$\alpha_{CO2/CH4}$	Ref.
DDR	HT d	Tube	74.73	700 ºC, 5 h	~5	4	700	220	40
DDR	HT d	Tube	55.6	800 ºC, 5 h	2-3	2	3,000	200	39
DDR	HT d	Disk	2.54	700 ºC, 6 h	10	2	1,800	62	46
DDR	MV e	Tube	18.84	200 ºC, 96 h b	~1	2.4	4,700	190	2
DDR	HT d	Tube	31.4	200 ºC, 24 h b	~5	2	1,200	270	47
DDR	HT d	Hollow fiber	8.4	200 ºC, 96 h b	~5	2	350	500	38
SSZ-13	HT d	Hollow fiber	1.7	450 ºC, 80 h c	4-6	6	3,000	42	31
SSZ-13	HT d	Hollow fiber	1.42	550 ºC, 10 h	1.2±0.6	1	11,700	210	33
DDR	HT d	Hollow fiber (20 cm)	24	TMCT +CC	~5	3	1,400	487	This work
DDR	HT d	Hollow fiber (20 cm)	24	TMCT +CC	~5	3	890	1,172	This work
DDR	HT d	Hollow fiber (20 cm)	24	TMCT +CC	~5	3	1,420	843	This work

[a]: $\times 10^{-10}$ mol m^{-2} s^{-1} Pa^{-1}; [b-c]: ozone and oxygen were used as atmosphere; [d-e]: oven and microwave were used for heating.

Supplementary Table 7 CO_2/CH_4 separation performance of DD3R zeolite membrane before and after 2-year storage in air atmosphere.

Membrane	Num.	Separation performance			
		Feed	Pressure / bar	P_i / mol m^{-2} s^{-1} Pa^{-1}	α
As-synthesized	1	CO_2/CH_4	31	3×10^{-8}	43
	2	CO_2/CH_4	31	4.1×10^{-8}	51
2-year stored	1	CO_2/CH_4	31	3.08×10^{-8}	44
	2	CO_2/CH_4	31	4.2×10^{-8}	49

Supplementary References

1. Liu Z, Gao Q, Chen J, Deng J, Lin K, Xing X. Negative thermal expansion in molecular materials. *Chem Commun* **54**, 5164-5176 (2018).

2. Lu X, Yang Y, Zhang J, Yan Y, Wang Z. Solvent-Free Secondary Growth of Highly b-Oriented MFI Zeolite Films from Anhydrous Synthetic Powder. *J Am Chem Soc* **141**, 2916-2919 (2019).

3. Liu S*, et al.* Hydrothermal treatment of grass: a low-cost, green route to nitrogen-doped, carbon-rich, photoluminescent polymer nanodots as an effective fluorescent sensing platform for label-free detection of Cu(II) ions. *Adv Mater* **24**, 2037-2041 (2012).

4. Gies H. Studies on clathrasils. IX Crystal stmcture of deca-dodecasil 3R, the missing link between zeolites and clathrasils *Zeitschrift für Kristallographie-Crystalline Materials* **175**, 93-104 (1986).

5. Momma K, Izumi F. VESTA: a three-dimensional visualization system for electronic and structural analysis. *J Appl Crystallogr* **41**, 653-658 (2008).

6. Dolomanov OV, Bourhis LJ, Gildea RJ, Howard JAK, Puschmann H. OLEX2: a complete structure solution, refinement and analysis program. *J Appl Crystallogr* **42**, 339-341 (2009).

7. van den Bergh J, Zhu W, Gascon J, Moulijn JA, Kapteijn F. Separation and permeation characteristics of a DD3R zeolite membrane. *J Membr Sci* **316**, 35-45 (2008).

8. van den Bergh J, Tihaya A, Kapteijn F. High temperature permeation and separation characteristics of an all-silica DDR zeolite membrane. *Microporous Mesoporous Mater* **132**, 137-147 (2010).

9. van den Bergh J, Ban S, Vlugt TJH, Kapteijn F. Modeling the loading dependency of diffusion in zeolites: the relevant site model extended to mixtures in DDR-type zeolite. *J Phys Chem C* **113**, 21856-21865 (2009).

10. Jee SE, Sholl DS. Carbon dioxide and methane transport in DDR zeolite: Insights from molecular simulations into carbon dioxide separations in small pore zeolites. *J Am Chem Soc* **131**, 7896-7904 (2009).

11. Houde AY, Krishnakumar B, Charati SG, Stern SA. Permeability of dense (homogeneous) cellulose acetate membranes to methane, carbon dioxide, and their mixtures at elevated pressures. *J Appl Polym Sci* **62**, 2181-2192 (1996).

12. Wind JD, Paul DR, Koros WJ. Natural gas permeation in polyimide membranes. *J Membr Sci* **228**, 227-236 (2004).

13. Fuertes AB, Nevskaia DM, Centeno TA. Carbon composite membranes from Matrimid® and Kapton® polyimides for gas separation. *Microporous Mesoporous Mater* **33**, 115-125 (1999).

14. Lei L, Lindbråthen A, Hillestad M, Sandru m, Favvas EP, He X. Screening cellulose spinning parameters for fabrication of novel carbon hollow fiber membranes for gas separation. *Ind Eng Chem Res* **58**, 13330-13339 (2019).

15. Hou M*, et al.* Carbon molecular sieve membrane with tunable microstructure for CO_2 separation: Effect of multiscale structures of polyimide precursors. *J Membr Sci* **635**, 119541 (2021).

16. Qiu W, Leisen JE, Liu Z, Quan W, Koros WJ. Key features of polyimide-derived carbon molecular sieves. *Angew Chem Int Ed* **60**, 22322-22331 (2021).

17. Babu DJ*, et al.* Restricting lattice flexibility in polycrystalline metal-organic framework membranes for carbon capture. *Adv Mater* **31**, 1900855 (2019).

18. Rui Z, James JB, Kasik A, Lin YS. Metal-organic framework membrane process for high purity CO_2 production. *AIChE J* **62**, 3836-3841 (2016).

19. Wang Y*, et al.* A MOF glass membrane for gas separation. *Angew Chem Int Ed* **59**, 4365-4369 (2020).

20. Wang B*, et al.* Separation of light gas mixtures using zeolite SSZ-13 membranes. *Microporous Mesoporous Mater* **275**, 191-199 (2019).

21. Li S, Falconer JL, Noble RD. Improved SAPO-34 membranes for CO_2/CH_4 separations. *Adv Mater* **18**, 2601-2603 (2006).

22. Wu T, Wang B, Lu Z, Zhou R, Chen X. Alumina-supported AlPO-18 membranes for CO_2/CH_4 separation. *J Membr Sci* **471**, 338-346 (2014).

23. Robeson LM. The upper bound revisited. *J Membr Sci* **320**, 390-400 (2008).

24. Bos A, Pünt IGM, Wessling M, Strathmann H. Plasticization-resistant glassy polyimide membranes for CO2/CO4 separations. *Sep Purif Technol* **14**, 27-39 (1998).

25. Jiang L, Chung T-S, Li DF, Cao C, Kulprathipanja S. Fabrication of Matrimid/polyethersulfone dual-layer hollow fiber membranes for gas separation. *J Membr Sci* **240**, 91-103 (2004).

26. Syrtsova DA, Kharitonov AP, Teplyakov VV, Koops GH. Improving gas separation

properties of polymeric membranes based on glassy polymers by gas phase fluorination. *Desalination* **163**, 273-279 (2004).

27. Tin PS, Chung TS, Liu Y, Wang R, Liu SL, Pramoda KP. Effects of cross-linking modification on gas separation performance of Matrimid membranes. *J Membr Sci* **225**, 77-90 (2003).

28. Scholes CA, Stevens GW, Kentish SE. Membrane gas separation applications in natural gas processing. *Fuel* **96**, 15-28 (2012).

29. Carreon ML, Li S, Carreon MA. AlPO-18 membranes for CO_2/CH_4 separation. *Chem Commun* **48**, 2310-2312 (2012).

30. Carreon MA, Li S, Falconer JL, Noble RD. Alumina-supported SAPO-34 membranes for CO_2/CH_4 separation. *J Am Chem Soc* **130**, 5412-5413 (2008).

31. Kosinov N*, et al.* High flux high-silica SSZ-13 membrane for CO_2 separation. *J Mater Chem A* **2**, 13083-13092 (2014).

32. Yang S, Kwon YH, Koh D-Y, Min B, Liu Y, Nair S. Highly selective SSZ-13 zeolite hollow fiber membranes by ultraviolet activation at near-ambient temperature. *ChemNanoMat* **5**, 61-67 (2019).

33. Yang S, Chiang Y, Nair S. Scalable one-step gel conversion route to high-performance CHA zeolite hollow fiber membranes and modules for CO_2 separations. *Energy Technology* **7**, 1900494 (2019).

34. Karakilic P, Wang XR, Kapteijn F, Nijmeijer A, Winnubst L. Defect-free high-silica CHA zeolite membranes with high selectivity for light gas separation. *J Membr Sci* **586**, 34-43 (2019).

35. Yu L, Holmgren A, Zhou M, Hedlund J. Highly permeable CHA membranes prepared by fluoride synthesis for efficient CO_2/CH_4 separation. *J Mater Chem A* **6**, 6847-6853 (2018).

36. Wu T*, et al.* Influence of propane on CO_2/CH_4 and N_2/CH_4 separations in CHA zeolite membranes. *J Membr Sci* **473**, 201-209 (2015).

37. Wang M*, et al.* Ultrafast synthesis of thin all-silica DDR zeolite membranes by microwave heating. *J Membr Sci* **572**, 567-579 (2019).

38. Wang L, Zhang C, Gao X, Peng L, Jiang J, Gu X. Preparation of defect-free DDR zeolite membranes by eliminating template with ozone at low temperature. *J Membr Sci* **539**, 152-160 (2017).

39. Himeno S, Tomita T, Suzuki K, Nakayama K, Yajima K, Yoshida S. Synthesis and permeation

properties of a DDR-type zeolite membrane for separation of CO_2/CH_4 gaseous mixtures. *Ind Eng Chem Res* **46**, 6989-6997 (2007).

40. Tomita T, Nakayama K, Sakai H. Gas separation characteristics of DDR type zeolite membrane. *Microporous Mesoporous Mater* **68**, 71-75 (2004).

41. Sridhar S, Smitha B, Aminabhavi TM. Separation of Carbon Dioxide from Natural Gas Mixtures through Polymeric Membranes—A Review. *Sep Purif Rev* **36**, 113-174 (2007).

42. Tang X*, et al.* Fast synthesis of thin SSZ-13 membranes by a hot-dipping method. *J Membr Sci* **629**, 119297 (2021).

43. Kajihara K*, et al.* Twinning by Merohedry and Thermal Expansion of Zeolitic Clathrasil Deca-dodecasil 3R. *Inorg Chem* **59**, 5600-5609 (2020).

44. Park SH, Kunstleve RWG, Graetsch H, Gies H. The thermal expansion of the zeolites MFI, AFI, DOH, DDR, and MTN in their calcined and as synthesized forms. In: *Progress in Zeolite and Microporous Materials, Pts a-C* (ed^(eds Chon H, Ihm SK, Uh YS) (1997).

45. Lightfoot P, Woodcock DA, Maple MJ, Villaescusa LA, Wright PA. The widespread occurrence of negative thermal expansion in zeolites. *J Mater Chem* **11**, 212-216 (2001).

46. Howarth AJ*, et al.* Chemical, thermal and mechanical stabilities of metal-organic frameworks. *Nat Rev Mater* **1**, 15018 (2016).

47. Hayakawa E, Himeno S. Synthesis of a DDR-type zeolite membrane by using dilute solutions of various alkali metal salts. *Sep Purif Technol* **218**, 89-96 (2019).
